# Clinical Cases

**Published:** 1893-03

**Authors:** W. L. Morgan

**Affiliations:** Baltimore, Md.


### CLINICAL CASES.

W. L. Morgan, M. D., Baltimore, Md.

Miss Bessie B, aged twelve years, light hair, blue eyes, slender. Had measles eight years ago, suffered a great deal, never got well properly, has had a hoarse, rough cough ever since. Always taking cold at every provocation. Was frequently called to see her for colds. Always attacked in a different way; never twice alike.

March 20th, 1891.—Cough worse, rough, hoarse. Gave Spongia$^{60}$. The 28th called again. Cough better, but had bad headache, backache, tongue red, raw, and salt smarted tongue badly. Gave Nat-m.$^{cm}$.

May 12th.—Rough, hoarse cough again. Gave Spongia$^{200}$.

May 18th.—Tongue coated white, sore in the breast, rattling phlegm, difficult expectoration of white mucus. Gave Ant-tart.$^{60}$.

May 29th.—Called early in the morning and found measles out all over. Cough nearly stopped; feeling much better. Run an easy course. Has been in good health, no cough since.

John Dwier, age eleven years. Red hair, light complexion, rather delicate build, mild disposition. Called February 11th, 1892. High fever, sore throat, hoarse, rough cough. Could not make any diagnosis but found he had had measles four years before. Had not developed well. Has had the cough ever since. I suspected suppressed measles. Gave Puls.$^{60}$. Next day all symptoms appeared to be worse, especially cough and sore throat. I saw signs of measles on the soft palate. Gave Sac-lac. Next morning measles fully out. Run an easy course; was well soon. Two other children took measles from him. All got well without further trouble. John in better health ever since.

I report these two cases in addition to the four cases published in the *Advance* of April, 1891, as increased additional evidence that measles may remain in the system for an indefinite number of years and by judiciously following the indications for treatment may be re-developed and the patient restored to health, provided the destruction of tissue has not gone too far as in the latter stages of consumption caused by suppressed measles.

John D. Newman, age sixty-four years, medium height, railroad engineer for about forty years. Generally in good health. Suffering from indolent ulcers on both legs, front part from knee to ankle, for eight years; scaly, suppurating but little, leaving a purple base after healing, but reopening frequently, very sore to touch but not painful. The leading symptom for treatment was always feeling badly in a warm room and better in open air; great aversion to exercise, always wanting to lie quietly and not be disturbed.

April 18th, 1891.—Gave Bry.$^{200}$, and Sac-lac.

April 24th.—Feeling better. Continued Sac-lac.

October 30th.—Continued improvement. Continued Sac-lac.

November 14th.—No improvement. Gave Bry.$^{m}$, and Sac-lac.

November 29th.—Little improved. Gave Bry.$^{mm}$ (Skinner).

December 5th.—Sore all healed and color nearly all disappeared, but he had been on road again and had taken cold and returned home with typhoid fever. Bry. being indicated gave the 200th, which was the remedy indicated by symptoms of the fever. Made a good recovery and has been in good health ever since.

The same remedy appeared to be indicated in the dissimilar case of the same patient. In the latter case the 200th in two doses cured him and he still remains well.

April 9th, 1892.—Mrs. W., age forty-five years; weight, one hundred and twenty-five pounds; light complexion, native of Pennsylvania. Had suffered some years ago with some uterine and vesical trouble. The past two years had suffered with menorrhagia, after flowing for two and one-half months without intermission. Had resisted carefully selected treatment, also the treatment of a modern homœopathist and returned to me. Gave Ovi-mem.$^{dmm}$ (Swan). One dose 4 P. M. At 10 P. M. had intense itching from waist down with heat and feeling like measles when rubbed. Washed with warm water and soap, then it felt as if being burned but soon cooled off and slept well. Awoke as usual 9 A. M., frontal headache from brow to sinciput. Next day no itching, headache some, flow growing less and pale, otherwise well as usual. These symptoms were all new, had never experienced any of them before. Next day flow stopped. Since then great improvement in health.

June 16th.—Health continues excellent.

February 17th.—Was called to see Mrs. ——. Great pain in right hypogastric and inguinial region, swelling and soreness and bloody leucorrhœal discharge. I soon learned that she had had a miscarriage on the 10th; had been under the care of another physician, that he had done something to induce miscarriage but she did not know what; that she has suffered a great deal and was getting no better, and was sure that something was wrong. Gave Arnica$^{30}$.

February 18th.—No better. Calendula$^{30}$ with compress of same in solution of water. Continued through many changes of symptoms as well as remedies, but Calendula all the time to correspond to the best of my judgment. The swelling subsided and for several days there remained a hard object like a cord the size of a small finger to the feel but quite firm, extending from the fundus uteri parallel with and just above Poupart's ligament to a point just above the anterior crest of the ileum, thence a short curve upward as if with the ascending colon; the great emaciation of the patient rendered it easy to determine that this object was inside of the abdominal parietes; by daily examination it proved to be moving to the right and upward. On March 7th it had left the fundus uteri over an inch and the vaginal discharge had nearly ceased. An examination found vagina perfectly healthy, os opened half an inch, filled with clear mucus which was removed; found opening an inch deep, very red but not sore or bleeding. General health improved. March 28th the mysterious object had disappeared to the right and upward. Patient dismissed.

From this time everything went well with patient till April

23d. She came to my office appearing in a very uneasy state of mind, stating that while at stool in the morning she had passed with the stool a piece of something like a bone which she lost in the sink, and was followed by a something like a large worm which she had pulled from the anus with great pain and difficulty, and washed it and brought it for me to see what it was, and produced from her pocket a package containing a common sized flexible bougie with the ivory head taken off, which I supposed was the part that fell in the sink and was lost.

From this train of evidence I am led to conclude that the bougie had been used to produce abortion and not been removed at the delivery, and when the uterus contracted the end of the bougie engaged the inner opening of the right Fallopian tube and from the continual pressure and irritation caused inflammation and expansion of the tube, and it easily passed upward to the right extremity, and by inflammation and adhesions perforated the ascending colon and thence continued to move along with the colon to the anus, and passed out through the natural channel. It is still in my possession. The patient is now satisfied that if she gets pregnant she will have no operation to prevent its continuance.

